# Association of human leukocyte antigens‐DQB2/DPA1/DPB1 polymorphism and pulmonary tuberculosis in the Chinese Uygur population

**DOI:** 10.1002/mgg3.544

**Published:** 2019-01-01

**Authors:** Xue Wang, Xudong Cao, Wanjiang Zhang, Le Zhang, Lijun Lu, Xinyue Li, Saeed El‐Ashram, Jiangdong Wu, Chuangfu Chen

**Affiliations:** ^1^ Key Laboratory of Xinjiang Endemic and Ethnic Diseases Cooperated by Education Ministry with Xinjiang Province Shihezi University Shihezi China; ^2^ College of Life Science and Engineering Foshan University Foshan China; ^3^ Faculty of Science Kafrelsheikh University kafr El-Sheikh Egypt

**Keywords:** HLA‐DPA, HLA‐DPB, HLA‐DQB, susceptibility, tuberculosis, Xinjiang Uygur

## Abstract

**Background:**

Tuberculosis (TB) is the second‐leading cause of death globally. Genetic polymorphisms in human leukocyte antigens (HLA)‐DQB2, HLA‐DPA1, and HLA‐DPB1 may partly explain individual differences in TB susceptibility.

**Methods:**

We performed a hospital‐based case–control study to assess the genetic influence of single‐nucleotide polymorphisms (SNPs) in the HLA (HLA‐DPA, HLA‐DPB, and HLA‐DQB) on the development of TB. There were 248 TB‐infected cases and 340 healthy controls in this study.

**Results:**

The HLA‐DQB2 rs7453920 genotype GG was applied as the reference group, the GA genotype was related to a considerably magnified risk of TB (GA vs. GG: adjusted OR = 1.547, 95% CI = 1.039–2.304, *p* = 0.032). Nevertheless, the other two SNPs were not associated with TB risk. Stratified analyses suggested that tobacco was associated with an increased risk of TB in HLA‐DQB2 rs7453920 G>A.

**Conclusion:**

These results suggested that the functional HLA‐DQB2 rs7453920 G>A polymorphism may contribute to the genetic susceptibility to TB. Nevertheless, the results were based on a limited sample size, and larger well‐designed studies are expected to confirm these preliminary findings.

## 1 INTRODUCTION

Tuberculosis (TB), an infectious disease caused by mycobacterium TB (MTB) infection, is the second‐leading cause of death worldwide according to World Health Organization (WHO) Global TB report (2016). It has been estimated that one third of the global population is infected with MTB. Compared with other continents, more than 50% of TB cases in the world are estimated to be in Asia with China ranking second in the world for TB prevalence after India (Liberato, de Albuquerque Mde, Campelo, & de Melo, 2004; Odone et al., 2015).

The southern part of Xinjiang Uygur Autonomous Region in the Northwest China is the home to the Uygur people (a Turkic ethnic group) where they live in isolation from the Han people (an east Asian ethnic group and nation) (Wang, Ma, Han, Litifu, & Xue, 2018). TB in Xinjiang Uygur autonomous region was higher than that in other provinces (Wubuli et al., 2015), which proved that Xinjiang had a heavy burden of TB. In the rs1017281 site located in ASAP1 gene, G allele was associated with increased risk of TB in the Chinese Xinjiang Muslim population (Wang et al., 2018). Environment and social factors may contribute to the high prevalence of *Mycobacterium tuberculosis *in the Uygur people (Kalo, Kant, Srivastava, & Sharma, 2015; Narasimhan, Wood, Macintyre, & Mathai, 2013; Patterson, Drewe, Pfeiffer, & Clutton‐Brock, 2017). Moreover, previous evidence suggested that single‐nucleotide polymorphisms (SNPs) in the context of genetic factors may play a pivotal role in TB susceptibility (Rolandelli et al., 2018). Human leukocyte antigens (HLA) is defined as the major histocompatibility complex, which consists mainly of class I and class II (Allard et al., 2014; Leddon & Sant, 2010).

Human leukocyte antigen plays an important role in the acquired immunity by distinguishing between self and nonself (Hudson & Allen, 2016). Genomewide association studies (GWAS) have recently discovered a strong association between the HLA‐DP and HLA‐DQ variants and the outcome of the hepatitis B virus (HBV) infection in Japan, Korea, and China (Nishida et al., 2014; Okada et al., 2017; Xiang et al., 2016). The HLA‐DP genetic variants have been recognized to correlate with occult hepatitis B infection (Mardian et al., 2017), and it has been shown that HLA‐DQ polymorphisms as protective factors are connected to HBV‐related hepatocellular carcinoma (Gao et al., 2016). Additionally, HLA‐DP and HLA‐DQ variants are involved in the progression of other diseases, such as graft‐versus‐host disease and cervical cancer (Jia et al., 2016; Morishima et al., 2018). HLA genes encode molecules that are central to the host immune response, and variation in these genes likely predicts the outcome of infectious diseases (Crux & Elahi, 2017). Recently, Icelanders have reported that three variants situated on the class II HLA gene affected TB susceptibility in populations of European ancestry in a GWAS (Sveinbjornsson et al., 2016). Similarly, there is substantial evidence that variations in the HLA class I and II genes could determine the outcome of MTB infection (Saraav et al., 2016; Toyo‐Oka et al., 2017). A case–control study in Chinese Han population verified that HLA class II locus rs9272461 affects the susceptibility to pulmonary TB (PTB) (Miao et al., 2018). Moreover, a study showed that HLA‐DR^+^CD4^+^ T cells may contribute to disease‐associated inflammation by compromising regulatory T cells‐mediated suppression in PTB (Ahmed et al., 2018). HLA‐DRB1 alleles *01, *03, *11, *13, *07, and *15 were observed significantly rare in children with TB in comparison with healthy donors that may indicate their protective role in the development of the disease (Starshinova et al., 2018). According to the biological and pathologic effect of HLA‐DPA, HLA‐DPB, and HLA‐DQ, we hypothesize that these variant genes may account for the development of TB. Despite the proven role of HLA‐DP rs3077, HLA‐DP rs9277535, and HLA‐DQ rs7453920 polymorphisms in the devolvement of diseases like hepatitis B, no current evidence shows whether HLA‐DPA1 (OMIM: 142880), HLA‐DPB1 (OMIM: 142858), and HLA‐DQB2 (OMIM: 615161) genetic variants could influence the outcome of TB. In light of the biological and pathologic effect of HLA‐DPA, HLA‐DPB, and HLA‐DQ in disease immunity, we hypothesize that these variant genes play an important role in the development and susceptibility to TB. In this study, we investigated the potential relationship of HLA‐DPA1 rs3077, HLA‐DPB1 rs9277535, HLA‐DQB2 rs7453920 with the occurrence of PTB. We enrolled 248 PTB cases and 340 controls to analyze three SNPs in a Xinjiang Uygur population that may be associated with TB development.

## 2 MATERIALS AND METHODS

### 2.1 Ethical approval of the study protocol

We have complied with the world Medical Association Declaration of Helsinki regarding ethical conduct of research involving human subjects and/or animals. This hospital‐based case–control study was approved by the Review Board of Kashgar Pulmonary Hospital (Xinjiang, China). A written informed consent was obtained from all subjects who were recruited and interviewed for the study.

### 2.2 Cases and controls

A total of 588 Uygur Chinese subjects aged 16–90 years old without miscegenation (no mixed descendant in three generations) were selected from Kashgar population in the Xinjiang Uygur Autonomous Region of China. TB (*n* = 248) and non‐TB (*n* = 340) patients (case–control study) were recruited from Kashgar pulmonary hospital and the first people's hospital of Kashgar for a two‐year period. Cases were selected according to the national diagnostic criteria of China, with positive sputum smear or culture, or significant symptoms of typical PTB, chest radiography consistent with active disease, and a positive tuberculin skin test in case of negative sputum, smear or culture. Patients who previously had HIV, any autoimmune, chronic inflammatory or other disease conditions were excluded from the study. Controls (patients without PTB) and cases were recruited in the same period. Cases were included in the study after detecting clinical manifestations, examining peripheral blood samples, X‐ray images and matching the cases with sex and age.

Demographic and risk factor information was obtained from cases and control subjects using a pretested questionnaire. Then, 2 ml of venous blood was obtained from all subjects. Smokers were defined as people who smoked one cigarette per day for >1 year. Alcohol drinkers were defined as subjects who consumed ≥3 alcoholic drinks a week for >6 months.

### 2.3 Isolation of DNA and genotyping of HLA genes

Genomic DNA was extracted from peripheral blood leukocytes using QIAamp DNA Blood Mini Kit (Qiagen, Berlin, Germany) following manufacturer's instructions and the procedure used by El‐Ashram, Al Nasr, and Suo (2016). Multiplex polymerase chain reaction (M‐PCR)‐ligase detection reaction was used to genotype blood DNA for three SNPs in HLA‐DPA1 (GenBank: KJ901483.1; rs3077), HLA‐DPB1 (GenBank: KJ905775.1; rs9277535), HLA‐DQB2 (GenBank: KJ901487.1; rs7453920) on an ABI3730XL Sequence Detection System (Applied Biosystems, Foster City, CA, USA). Technical support was provided by the Shanghai Genesky Biotechnology Company.

Data analysis was analyzed using GeneMapper Software v4.1 (AppliedBiosystems, USA). DNA sequencing was used to validate the genotype results by LDR. Results of LDR were identical with the results of subsequent sequencing for the randomly selected DNA samples from each genotype. Blood samples were collected from patients into ethylenediamine tetra‐acetic acid vacutainers.

Genomic DNA was isolated from whole human blood with the QIAamp DNA Blood Mini Kit (Qiagen, Berlin, Germany). Sample DNA (10 ng) was amplified by M‐PCR (Reference) according to the manufacturer's recommendations. The SNP genotyping work was carried out using a custom‐by‐design 48‐Plex SNPscan™ Kit (Genesky Biotechnologies Inc., Shanghai, China) according to the manufacturer's instructions. This kit was developed according to patented SNP genotyping technology by Genesky Biotechnologies Inc., based on double ligation and multiplex fluorescence PCR. For quality control, repeat analyses were performed for 4% of randomly selected samples.

### 2.4 Statistical results

Differences in the distributions of demographic characteristics, selected variables, and genotypes of the HLA‐DPA1 rs3077 G>A, HLA‐DPB1 rs9277535 G>A, and HLA‐DQB2 rs7453920 G>A variants in the cases and controls were evaluated using the chi‐square test. The relations between HLA‐DPA1 rs3077 G>A, HLA‐DPB1 rs9277535 G>A, HLA‐DQB2 rs7453920 G>A genotypes and infection of *M. tuberculosis* were estimated by computing the odds ratios (ORs) and their 95% confidence intervals (CIs) using logistic regression analyses for crude ORs and adjusted ORs, by adjusting for age, sex, smoking, and drinking status. The Hardy–Weinberg equilibrium (HWE) was tested by a goodness‐of‐fit chi‐square test to compare the observed genotype frequencies to the expected ones among the control subjects. SPSS 17.0 was utilized for data management and statistical analyses.

Chi‐square test was used to compute goodness‐of‐fit to the HWE as well as genotypes and allele distributions between PTB and controls. Logistic regression analysis was performed to investigate the association between related SNPs and LOAD risk after adjustment for age, sex, alcohol, and tobacco use. Statistical significance refers to two‐sided *p* values of <0.05.

## 3 RESULTS

### 3.1 Characteristics of the study population

Demographic features of cases and controls are shown in Table 1. No significant differences regarding sex or alcohol use between the cases and the controls as suggested by the chi‐square tests (*p = *0.767 and *p = *0.936, respectively). However, we observed significant differences (*p = *0.041 and *p* = 0.000, respectively) in the distributions of demographic when tobacco smoking and age were considered. Data obtained from the three genotyped SNPs were shown in Table 2.

**Table 1:** Distribution of selected demographic variables and risk factors in Tuberculosis cases and controls

Variable	Cases (*n* = 248)		Controls (*n* = 340)		*p* a
	*n*	%	*n*	%	
Age (years)					
<60	108	43.5	282	82.9	**0.000**
≥60	140	56.5	58	17.1	
Sex					
Male	111	44.8	148	43.5	0.767
Female	137	55.2	192	56.5	
Smoking					
Ever	69	27.8	70	20.6	**0.041**
Never	179	72.2	270	79.4	
Alcohol use					
Never	197	79.4	271	79.7	0.936
Ever	51	20.6	69	20.3	

a Two‐sided chi‐square test; bold values are statistically significant (*p* < 0.05).

**Table 2:** Primary information for three genotyped single‐nucleotide polymorphisms (SNPs)

Genotyped SNPs	chr	Location	Test for HWE (*p* value)	MAF in our controls (*n* = 340)		MAF (Hapmap‐HCB)		MAF (Hapmap‐CEU)		Genotyping value (%)
rs3077 G>A	6	/	0.4081	0.305 (0.695)	G(A)	0.369	A	0.155	G	98.936
rs7453920 G>A	6	3’UTR	0.6789	0.27	A	0.157	A	0.478	A	98.936
rs9277535 G>A	6	3’UTR	0.7517	0.318	G	0.438	A	0.261	G	98.632

Notes

HWE: Hardy–Weinberg equilibrium; MAF(Hapmap‐CEU): minor allele frequency in European population; MAF(Hapmap‐HCB): minor allele frequency in Chinese population.

For the three SNPs, the genotype polymorphism ranged from 98.632% to 98.936% in all 588 samples. Furthermore, analysis of concordance rates by the random double‐blind method was 100%. Minor allele frequency of three genotyped SNPs in our controls (the Uygur Chinese) was less than that in the European population but higher than the Chinese Han population. The observed genotype frequencies for these three polymorphisms in the controls were consistent with HWE (Table 2).

### 3.2 Association of the three polymorphisms and risk of PTB

The genotype distributions of rs3077 G>A, rs7453920 G>A, and rs9277535 G>A in both populations are shown in Table 3. In the single locus analysis, the genotype frequencies of HLA‐DQB2 rs7453920 G>A were 48.8% (GG), 44.4% (GA), and 6.9% (AA) in the case subjects and 60.6% (GG), 29.7% (GA), and 9.7% (AA) in the control individuals. The two populations were considerably different regarding genotype polymorphism (*p* = 0.032). When the HLA‐DQB2 rs7453920 GG homozygote genotype was considered as the reference group, the GA genotype was significantly associated with an increased risk of TB (GA vs. GG: adjusted OR = 1.547, 95% CI = 1.039–2.304, *p* = 0.032). However, when the HLA‐DQB2 rs7453920 GG homozygote genotype was considered as the reference group, the AA genotype was not a risk of TB (AA vs. GG: adjusted OR = 0.933, 95% CI = 0.474–1.835, *p* = 0.841).

**Table 3:** Logistic regression analyses of associations between HLA‐DPA1 rs3077 G>A, HLA‐DQB2 rs7453920 G>A and HLA‐DPB1 rs9277535 G>A polymorphisms and risk of Tuberculosis

Genotype	Cases (*n* = 248)		Controls (*n* = 340)		Crude OR (95% CI)	*p*	Adjusted OR^a^ (95% CI)	*p*
	*n*	%	*n*	%				
rs3077 G>A								
GG	24	9.7	24	7.1	1.00		1.00	
GA	103	41.5	153	45	0.673 (0.363–1.250)	0.21	0.635 (0.322–1.251)	0.189
AA	121	48.8	163	47.9	0.742 (0.402–1.370)	0.341	0.726 (0.371–1.421)	0.35
GA+AA	145	58.5	316	92.9	0.709 (0.392–1.280)	0.254	0.682 (0.357–1.302)	0.246
GG+GA	127	51.2	177	52.1	1.00		1.00	
AA	121	48.8	163	47.9	1.035 (0.746–1.436)	0.839	1.063 (0.741–1.524)	0.741
rs7453920 G>A								
GG	121	48.8	206	60.6	1.00		1.00	
GA	110	44.4	101	29.7	1.854 (1.305–2.634)	**0.01**	1.547 (1.039–2.304)	**0.032**
AA	17	6.9	33	9.7	0.877 (0.469–1.641)	0.682	0.933 (0.474–1.835)	0.841
GA+AA	127	51.2	134	39.4	1.614 (1.159–2.246)	0.005	1.392 (0.961–2.017)	0.081
GG+GA	231	93.1	307	90.3	1.00		1.00	
AA	17	6.9	33	9.7	0.685 (0.372–1.259)	0.223	0.796 (0.411–1.540)	0.498
rs9277535 G>A								
GG	29	11.7	32	9.4	1.00		1.00	
GA	97	39.1	150	44.1	0.714 (0.406–1.254)	0.241	0.662 (0.356–1.228)	0.191
AA	122	49.2	158	46.5	0.852 (0.489–1.485)	0.572	0.849 (0.462–1.580)	0.592
GA+AA	219	88.3	308	90.6	0.785 (0.461–1.335)	0.371	0.756 (0.422–1.353)	0.346
GG+GA	126	50.8	182	53.5	1.00		1.00	
AA	122	49.2	158	46.5	1.115 (0.804–1.548)	0.514	1.179 (0.820–1.693)	0.374

Notes

Both rs3077 G>A and rs9277535 G>A genotypes were not statistically significant. However, only GA genetype was meaningful in the rs7453920 G>A variant,and others were not statistically reliable markers for TB.

HLA‐DQB2 GenBank: KJ901487.1; HLA‐DPA1 GenBank: KJ901483.1; HLA‐DPB1 GenBank: KJ905775.1.

a Adjusted for age, sex, smoking status and alcohol consumption: bold values are statistically significant (*p* < 0.05).

In the recessive model, when the HLA‐DQB2 rs7453920 GG/GA genotype was applied as the reference group, there was no association between the AA genotype and the risk of TB (adjusted OR = 0.796, 95% CI = 0.411–1.540, *p* = 0.498). In the dominant model, compared to the HLA‐DQB2 rs7453920 GG genotype, the HLA‐DQB2 rs7453920 GA/AA variants were related to an increased risk of TB (adjusted OR = 1.392, 95% CI = 0.961–2.017, *p* = 0.081; Table 3). However, in the genotype distributions of rs3077 G>A and rs9277535 G>A, there was no difference between the two populations. Logistic regression analyses suggested that there was no association between the two polymorphisms and the risk of TB (Table 3).

### 3.3 Stratification analyses of rs7453920 G>A polymorphisms and risk of TB

Based on sex, tobacco, and alcohol consumption, the stratification analyses were carried out to assess the effects of rs7453920 G>A genotype on the risk of TB. When the HLA‐DQB2 rs7453920 GG homozygote genotype was applied as the reference group, the GA genotype was significantly associated with an increased risk of TB more than tobacco (GA vs. GG: adjusted OR = 2.385, 95% CI = 1.439–3.954, *p* = 0.001). However, when the HLA‐DQB2 rs7453920 GG homozygote genotype was applied as the reference group, the AA genotype was not associated with the risk of TB more than tobacco (AA vs. GG: adjusted OR = 1.146, 95% CI = 0.520–2.527, *p* = 0.735). In the recessive model, when the HLA‐DQB2 rs7453920 GG/GA genotype was applied as the reference group, the association between the AA genotype and the risk of TB was less than tobacco (adjusted OR = 1.989, 95% CI = 1.254–3.154, *p* = 0.003). In the dominant model, compared to the HLA‐DQB2 rs7453920 GG genotype, the HLA‐DQB2 rs7453920 GA/AA variants were associated with an increased risk of TB more than tobacco (adjusted OR = 0.862, 95% CI = 0.402–1.850, *p* = 0.704; Table 4). The other two polymorphisms were not associated with tobacco.

**Table 4:** Stratified analyses between HLA‐DQB2 rs7453920 G>A polymorphisms and Tuberculosis risk by sex, age, smoking status, and alcohol consumption

Variable	rs7453920 G>A (case/control)^a^				Adjusted OR^b^ (95% CI); *p*				
	GG	GA	AA	GA+AA	GG	GA	AA	GA+AA	AAvs (GA+GG)
Sex									
Male	52/96	52/37	7/15	59/52	1	1.824 (0.986–3.374); *p*: 0.055	0.829 (0.310–2.218); *p*: 0.709	1.501 (0.859–2.623); *p*: 0.154	0.685 (0.260–1.801); *p*: 0.442
Female	69/110	58/64	10/18	68/82	1	1.620 (0.925–2.837); *p*: 0.092	1.199 (0.460–3.127); *p*: 0.710	1.528 (0.901–2.590); *p*: 0.116	0.990 (0.392–2.502); *p*: 0.983
Age									
<60	50/180	49/74	9/28	58/102	1	2.138 (1.285–3.556); *p*: 0.003	1.101 (0.477–2.542); *p*: 0.821	1.843 (1.147–2.962); *p*: 0.012	0.816 (0.363–1.837); *p*: 0.624
≥60	71/26	61/27	8/5	69/32	1	1.191 (0.579–2.449); *p*: 0.634	0.461 (0.130–1.635); *p*: 0.230	1.013 (0.517–1.984); *p*: 0.970	0.432 (0.125–1.496); *p*: 0.186
Smoking status									
Never	30/28	37/36	2/6	38/42	1	0.841 (0.410–1.724); *p*: 0.636	0.317 (0.410–1.724); *p*: 0.184	0.760 (0.377–1.529); *p*: 0.441	0.346 (0.066–1.813); *p*: 0.209
Ever	91/178	73/65	15/27	88/92	1	2.385 (1.439–3.954); *p*: 0.001	1.146 (0.520–2.527); *p*: 0.735	1.989 (1.254–3.154); *p*: 0.003	0.862 (0.402–1.850); *p*: 0.704
Alcohol consumption									
Never	97/170	87/73	13/28	100/101	1	1.949 (1.215–3.127); *p*: 0.006	0.778 (0.347–1.748); *p*: 0.544	1.602 (1.034–2.482); *p*: 0.035	0.816 (0.280–1.357); *p*: 0.229
Ever	24/36	23/28	4/5	27/33	1	0.853 (0.367–1.979); *p*: 0.711	1.303 (0.303–5.592); *p*: 0.722	0.925 (0.422–2.031); *p*: 0.847	1.377 (0.331–5.725); *p*: 0.660

Notes

HLA‐DQB2 GenBank: KJ901487.1.

a The genotyping was successful in 248 (100%) tuberculosis cases, and 340 (100%) controls for HLA‐DQB2 rs7453920 G>A.

b Adjusted for age, sex, smoking status, and alcohol consumption (besides stratified factors accordingly) in a logistic regression model.

## 4 DISCUSSION

This study investigated the association between gene polymorphisms in HLA‐DPA, HLA‐DPB, and HLA‐DQB genes and susceptibility to TB in the hospital‐based case–control study. We confirmed the association of HLA‐DQB2 rs7453920 G>A with risk of TB. Multivariable logistic analysis revealed the association between rs7453920 GA genotype and an increased risk of TB. As mentioned in the literature, GA genotype of SNP rs2069837 A>G located within IL‐6 was associated with PTB, while GG genotype did not show any association with TB (Wu et al., 2018). Prior studies have noted the relationship between CT genotype of TNF‐857 and spinal TB, while TT genotype did not correlate with spinal TB (Zheng et al., 2018). Our research shows that heterozygous genotypes are meaningful; however, a number of studies have shown a significant evidence of homozygosity. The reasons may be ascribed to the following: (a) HLA function is extremely complicated and polymorphic, and GA phenotype alone may not be sufficient to be a key location for TB‐susceptible polymorphism; (b) HLA polymorphisms tend to be highly linked and haplotypic; and (c) due to HLA polymorphism, different ethnic groups in different geographic regions may have different TB outcomes. However, the rs3077 G>A and rs9277535 G>A polymorphisms were not associated with the risk of TB. To our knowledge, this is the first study, which has shown an association between HLA‐DQB2 rs7453920 SNP and an increased risk of TB in the Uygur population.

Previous studies from Ghana and Gambia detected SNP rs4331426 located on chromosome 18q11.2 and showed that this SNP was associated with TB susceptibility (Thye et al., 2010), while rs2057178 on chromosome 11p13 was identified as protective factor in TB patients (Thye et al., 2012). Furthermore, a previous study in China has also shown that SNP rs4331426 plays a role in susceptibility to TB (Wang et al., 2013). In addition, furthermore, a study in Iran demonstrated that HLA‐DRB1*07 and HLA‐DQA1*0101 could be the predisposing alleles while HLA‐DQA1*0301 and 0501 might play a protective role in TB patients (Amirzargar et al., 2004). A study in Kazakhstan found that HLA‐DQA1*03:02, HLA‐DRB1*08:01, and DRB1*08:03 were more frequent in patients carrying drug‐resistant TB, and a potential association between certain HLA alleles and TB was shown in the Kazakh population (Kuranov et al., 2014). In Koreans, a significant interaction was observed between HLA‐DQB1* 0601 allele and susceptibility to TB (Hong et al., 2007). Furthermore, a significant interaction between the less common DQB1* 0503 HLA class II allele associated with TB in Cambodia was also shown (Goldfeld et al., 1998). This hospital‐based case–control study investigated the associations of HLA‐DPA1 rs3077, HLA‐DPB1 rs9277535, and HLA‐DQB2 rs7453920 polymorphisms with the risk of PTB in the Chinese Uygur populations. Our multivariable logistic analysis revealed that HLA‐DQB2 rs7453920 was related to TB in the Uygur population. Noticeably, unlike other case–control studies, despite the strict selection criteria, the age difference in this study was significant, which made this study seemingly awkward.

In the stratification analysis, not drinking alcohol was a risk factor while alcohol consumption had no effect on the occurrence of TB. This meaningfulness of these results is unclear. However, this data may be biased toward nonconsumption of alcohol most the Uygur people do not drink alcohol because of religious reasons. Moreover, our controls were more than cases. Based on gender, ethnic groups, geographic locations, and other factors, genotype frequency distribution could diverge considerably. Besides, sample size, participant sources, inclusion and exclusion criteria, and different technical factors may also generate different results.

In the end, we have to address several limitations of this case–control study: First, the study populations/participants in this study were exclusively recruited in the hospital, which may have not been representative of the general population. Second, statistical power of our study was restricted by the limited sample size. Furthermore, larger studies are needed to confirm our findings, especially randomized clinical studies on the Uygur populations. Third, a comprehensive understanding of HLA genetic variability may have been limited because this study was biased toward only three gene targets. To distinctly and fully explore HLA gene variations that associated with the susceptibility to TB. Comprehensively, further fine mapping studies will be needed massively. Moreover, further analyses are also required to elaborate the mechanisms how the gene might specifically affect TB progression.

All in all, our study offers significant evidences that polymorphism of HLA‐DQB2 rs7453920 G>A may increase the risk of TB, and smoking is an independent risk factor for TB. This new information of host response to MTB infection can help to discover new diagnostic markers, identify risk populations and new treatment strategies. It is considered to be a breakthrough in TB prevention and treatment for selection of candidate genes and detection of polymorphic loci. Investigating the Xinjiang Muslim population susceptibility gene may provide a way for Xinjiang Uygur Autonomous Region to control TB. Future larger studies should be carried out to confirm the current primary findings in the Uygur populations.

## ETHICS APPROVAL

This hospital‐based case–control study was approved by the Review Board of Kashgar Pulmonary Hospital (Xinjiang, China).

## DISCLOSURES

The authors declare that they have no conflict of interests.
